# Beecher on His Love for Nature

**Published:** 1874-04

**Authors:** 


### BEECHER ON HIS LOVE FOR NATURE.

Oh, let me tell you a little bit about myself once in a while. I wouldn't take all the books of the Alexandrian library for the comfort I get out of nature. Nay, I had almost said I would rather lose my Bible than my world. There is no sunlight that does not speak to me of God. I sit down on the hillside in the summer afternoon and the grasshopper jumps over me, freshman like, jumping first and looking where he will land afterward. I sit so still that the birds forget me, and sing as though I was not there. The ants creep all over me. I am in fellowship with all. I am never so near to Him. This earth is a cathedral, whose windows are painted with rare beauty. Every day is a leaf in God's outside Bible. I did not once enjoy all this, but I owe to Ruskin more than to any theologian. Eyes I had but saw not, ears I had but didn't hear. I have become Hebraized. I have gone back to the noble stock, to the people who learned to discover the invisible God by the use of things seen.

